# Single-cell transcriptional profiling reveals developmental dynamics of longissimus dorsi muscle in adult and Juvenile bactrian camels

**DOI:** 10.3389/fphys.2025.1697432

**Published:** 2025-12-08

**Authors:** Baojun De, Yiyi Liu, Lu Li, Fanhua Meng, Yuwen Liu, Ting Jia, Yuting Chen, Chunxia Liu, Shenyuan Wang, Tao Li, Hongmei Xiao, Fang Wan, Yingchun Liu, Wenlong Wang, Huanmin Zhou, Wenguang Zhang, Xin Wen, Jie Gong, A. Naer, Hongmei Bao, Qian Song, HaSi Chaolu, Hai Long, Yanru Zhang, Junwei Cao

**Affiliations:** 1 College of Life Sciences, Inner Mongolia Agricultural University, Hohhot, China; 2 Inner Mongolia Key Laboratory of Biomanufacturing, Hohhot, China; 3 Inner Mongolia Endemic Livestock Biotechnology Innovation Team, Hohhot, China; 4 Research Centre for Animal Genome, Agricultural Genomics Institute at Shenzhen, Chinese Academy of Agricultural Sciences, Shenzhen, China; 5 College of Veterinary Medicine, Inner Mongolia Agricultural University, Hohhot, China; 6 Inner Mongolia Autonomous Region Agricultural and Animal Husbandry Technology Promotion Center, Hohhot, China; 7 School of Medicine, Hainan Vocational University of Science and Technology, Haikou, China; 8 Sunite Left Banner Animal Husbandry Workstation, Xilin Gol, China

**Keywords:** bactrian camel, skeletal muscle development, single-cell RNA sequencing, fibro-adipogenic progenitors, muscle fiber type

## Abstract

**Introduction:**

The longissimus dorsi muscle of Bactrian camels holds significant biological and economic value. However, the cellular heterogeneity, lineage differentiation patterns, and intercellular communication mechanisms underlying its skeletal muscle development remain unclear, which has hampered the advancement of precise regulation of camel meat quality traits and genetic improvement of the breed. Accordingly, there is an urgent need to elucidate the developmental regulatory mechanisms of this muscle tissue at the single-cell level.

**Methods:**

In this study, longissimus dorsi muscle tissues from 4-day-old (juvenile) and 5-year-old (adult) Bactrian camels were selected as research subjects. Integrated single-nucleus RNA sequencing (snRNA-seq) was employed to obtain gene expression data, which was coupled with Monocle2 pseudotime analysis, CellChat-based intercellular communication dissection, Gene Ontology (GO) enrichment analysis, and C2C12 cell functional validation experiments to conduct a systematic investigation into the cellular characteristics and developmental mechanisms of the Bactrian camel longissimus dorsi muscle.

**Results:**

A total of 14 cell clusters were identified, and the cellular composition of muscle tissues differed significantly between age groups—juvenile camel muscles were enriched with proliferative cell populations such as muscle satellite cells (MuSCs) and fibroblast-like progenitor cells (FAPs), while adult camel muscles were dominated by mature type IIX/IIA fast-twitch muscle fibers. Further analysis revealed that MuSCs exhibited bidirectional differentiation potential towards type I slow-twitch muscle fibers and type IIA/IIX fast-twitch muscle fibers, and the PCDH7 gene was found to promote myogenic differentiation. Additionally, four FAP subpopulations were characterized, among which the MME^+^ FAP subpopulation was closely associated with intramyocellular fat (IMF) deposition.

**Discussion:**

This study, for the first time, constructed a single-cell atlas and intercellular communication network of the Bactrian camel longissimus dorsi muscle, uncovered the key regulatory mechanisms governing its skeletal muscle development, and identified functionally important regulatory targets such as PCDH7. These findings not only provide a theoretical basis for the precise improvement of camel meat quality but also laid the groundwork for in-depth investigations into the adaptive evolutionary mechanisms of camel skeletal muscle.

## 1 Introduction

The Musculus longissimus dorsi is one of the skeletal muscles that holds both significant economic value and biological importance in animal husbandry. In livestock, this muscle serves as a core structure for maintaining spinal stability and transmitting locomotor force; post-slaughter, it constitutes the most valuable meat-cut portion. For meat-producing animals such as cattle, sheep, and pigs, the economic significance of the Musculus longissimus dorsi is particularly prominent (Cheng et al., 2015), and its developmental status is a key indicator for evaluating carcass meat yield and economic value. Additionally, the intramyocellular fat (IMF) content in this region significantly influences the premium pricing of meat products and eating quality (e.g., tenderness, juiciness, and flavor characteristics) (Van Ba et al., 2016). The Musculus longissimus dorsi has become a focal point in multi-omics research, encompassing data systems such as genomics, transcriptomics, proteomics, and metabolomics. Bactrian camels are not only crucial transportation tools in arid regions but also key food sources. Their multipurpose traits—providing meat, milk, leather, and more—significantly enhance their economic value (Kadim et al., 2018). However, dedicated research on the Musculus longissimus dorsi of Bactrian camels remains relatively scarce. Looking ahead, especially in the context of global climate change challenges, expanding research focus to extremophile-adapted species like camels has become particularly urgent. Notably, the interaction between myogenic cells and adjacent cell types in the longissimus dorsi muscle of Bactrian camels has not been fully explored. Therefore, in-depth analysis of the tissue’s cellular composition, functional heterogeneity, and molecular regulatory mechanisms will facilitate the establishment of precise genetic selection technologies, thereby directly improving camel meat production performance and economic benefits. Currently, the application of single-cell RNA sequencing (scRNA-seq) technology enables us to analyze transcriptomic characteristics at the single-cell level across different developmental stages. Compared with traditional transcriptomic methods (e.g., whole-embryo transcriptome sequencing and bulk RNA sequencing) (Mu et al., 2019), this technology offers higher resolution in gene expression analysis, particularly excelling in detecting cell types and their gene expression profiles (The Tabula Muris Consortium et al., 2018). A recent study analyzed 483 samples from various human tissues, constructing a cell atlas and gene expression profile covering nearly all human tissues (Xu D. et al., 2023). Similar studies have been conducted in numerous species, including mice, cattle, maize, and *Arabidopsis thaliana* (Jin L. et al., 2021; Potter, 2018; Jovic et al., 2022; Jones et al., 2022; Tabula Muris Consortium, 2020). In this study, we employed scRNA-seq technology to reveal the transcriptional regulatory dynamics of skeletal muscle development in Bactrian camels at the single-cell level. We analyzed samples of the Musculus longissimus dorsi from juvenile and adult Bactrian camels, constructing a high-resolution gene expression atlas. The results characterize myoblasts and their adjacent cell types across the two developmental stages, and clarify the developmental trajectory, cell state transitions, and intercellular communication networks of myoblasts. We also identified key factors regulating these processes and delineated the signaling pathways involved in the development of the Bactrian camel’s Musculus longissimus dorsi. This work not only provides valuable resources for understanding the molecular and cellular mechanisms of skeletal muscle development but also lays a foundation for research on the regulation of meat quality traits in Bactrian camels.

## 2 Materials and methods

### 2.1 Animals

Samples of the longissimus dorsi muscle from Sunite Bactrian camels were collected from Sonid Left Banner, Inner Mongolia. Two female individuals were sampled at each of two developmental stages: juvenile (4 days old) and adult (5 years old). All Bactrian camels were fasted overnight before euthanasia. The rearing and use of experimental animals fully complied with local animal welfare laws, guidelines, and policies.

### 2.2 Single-cell RNA sequencing data

Processing Raw 10× Genomics single-cell RNA sequencing (scRNA-seq) data were aligned to the reference genome from the ENSEMBL database (Release 104), and quantitative analysis was performed using Cell Ranger (v6.0.2) (Zheng et al., 2017). Subsequent bioinformatics analyses were implemented with the Seurat package (v4.0.5) (Stuart et al., 2019). During quality control, the R function ‘isOutlier’ was used to automatically identify and remove low-quality cells, including cells with too few detected genes, too low total reads, and abnormal mitochondrial-to-nuclear gene ratios (Luecken and Theis, 2019). For data preprocessing, the ‘NormalizeData’ and ‘ScaleData’ functions were applied to standardize and normalize the count matrix. Based on the top 2000 highly variable genes, Principal Component Analysis (PCA) was performed using the ‘RunPCA’ function (Hafemeister and Satija, 2019). To reduce the impact of background noise, significant principal components were screened according to the P-values calculated by ‘ScoreJackStraw’. Finally, cell clustering analysis was conducted using the ‘FindClusters’ function, and cells were projected onto a two-dimensional space for visualization via the ‘RunTSNE’ or ‘RunUMAP’ function (Butler et al., 2018).

### 2.3 Integrated analysis of single-cell RNA sequencing data

Before integrating multiple scRNA-seq datasets from different developmental stages, we first evaluated batch effects in the data (Yang et al., 2023). Preliminary analysis revealed that inter-sample differences constituted the most prominent source of technical variation, which was manifested by the tendency of cells to cluster by sample origin in dimensionality reduction visualizations (Xu J. et al., 2023). To eliminate such batch effects, we used the Harmony package (v0.1.0) for data integration and correction (Kang et al., 2023). Subsequently, the FindMarkers function in the Seurat toolkit was employed to identify marker genes for each cell cluster (Hao et al., 2024). Based on these marker genes and known cell type-specific markers, we performed manual annotation of cell clusters. Cell clusters with similar expression patterns were merged to obtain the final cell type annotation results.

### 2.4 Trajectory analysis of single-cell RNA sequencing data

Inference of cell developmental trajectories was conducted using the Monocle2 package (v2.20.0) (Qiu et al., 2017a). This analysis integrated scRNA-seq data to gain a more comprehensive understanding of the cell fate determination process. The analysis workflow mainly included the following steps: first, marker genes between different cell types were identified through differential expression analysis; second, the DDRTree algorithm (Qiu et al., 2017b) was used for dimensionality reduction of high-dimensional data and construction of a pseudotime-based cell developmental trajectory; finally, since Monocle2 requires manual specification of the developmental starting point, we set the muscle satellite cell (MuSC) stage as the trajectory origin by adjusting the root_state parameter, and the orderCells function was used to recalculate the temporal relationship of cells.

### 2.5 Detection of PCDH7 gene expression

C2C12 cells transfected with the PCDH7 overexpression lentiviral vector (overexpression-PCDH7), PCDH7 small interfering RNA (si-PCDH7), and empty lentiviral vector were separately seeded into 6-well plates. When the cell confluency reached 90%–100%, the medium was replaced with DMEM differentiation medium containing 2% horse serum to induce myogenic differentiation. Cell samples were collected on Days 0, 3, 5, and 7 post-induction. Total RNA was extracted, and quantitative real-time PCR (qRT-PCR) was used to detect the expression levels of the myogenic marker genes MYOD and MYOG.

### 2.6 Cell communication analysis

Quantitative analysis of intercellular communication networks was performed using the CellChat package (v1.1.3) (Jin S. et al., 2021). Given the limited annotation information for receptor-ligand pairs in the Bactrian camel genome, homologous human genes were selected for analysis in this study. The specific workflow was as follows: first, a CellChat object was constructed using the ‘createCellChat’ function, with normalized single-cell transcriptomic data imported. Subsequently, the ‘computeCommunProb’ and ‘computeCommunProbPathway’ functions were used to calculate and infer intercellular communication probabilities. To evaluate the topological characteristics of the communication network, the ‘netAnalysis_computeCentrality’ function was employed to calculate network centrality scores; meanwhile, the ‘netAnalysis_contribution’ function was used to analyze the contribution of each ligand-receptor pair within signaling pathways. Visualization of all cell communication networks was performed using built-in visualization functions of the CellChat package.

### 2.7 Gene Ontology (GO) enrichment analysis

GO enrichment analysis was conducted using the clusterProfiler package (v4.0) (Xu et al., 2024). Due to limitations in Bactrian camel genome annotation data, target genes were mapped to their human homologs, and GO term enrichment analysis was performed using the ‘enrichGO’ function in the org.Hs.eg.db database. To control the false positive rate in multiple testing, P-values were adjusted using the BenjaminiHochberg (BH) method.

## 3 Results

### 3.1 Identification of cellular heterogeneity during bactrian camel skeletal muscle development using scRNA-seq

To construct a cell atlas of the Bactrian camel longissimus dorsi muscle across its two developmental stages, longissimus dorsi muscle tissues were obtained from both juvenile and adult Bactrian camels in this study. For the longissimus dorsi muscle at each developmental stage, multi-region sampling (3 locations) was performed and samples were pooled to eliminate local tissue heterogeneity. The resulting pooled tissues underwent subsequent processing for single-cell isolation, and single-nucleus suspension samples were obtained via droplet-based scRNA-seq technology. A total of 34,325 high-quality single cells were captured (18,000 in the juvenile group and 16,325 in the adult group) for sequencing analysis. Based on the systematic analysis of differentially expressed genes (DEGs), we deeply revealed the molecular characteristics of each cell subpopulation.

**FIGURE 1:**
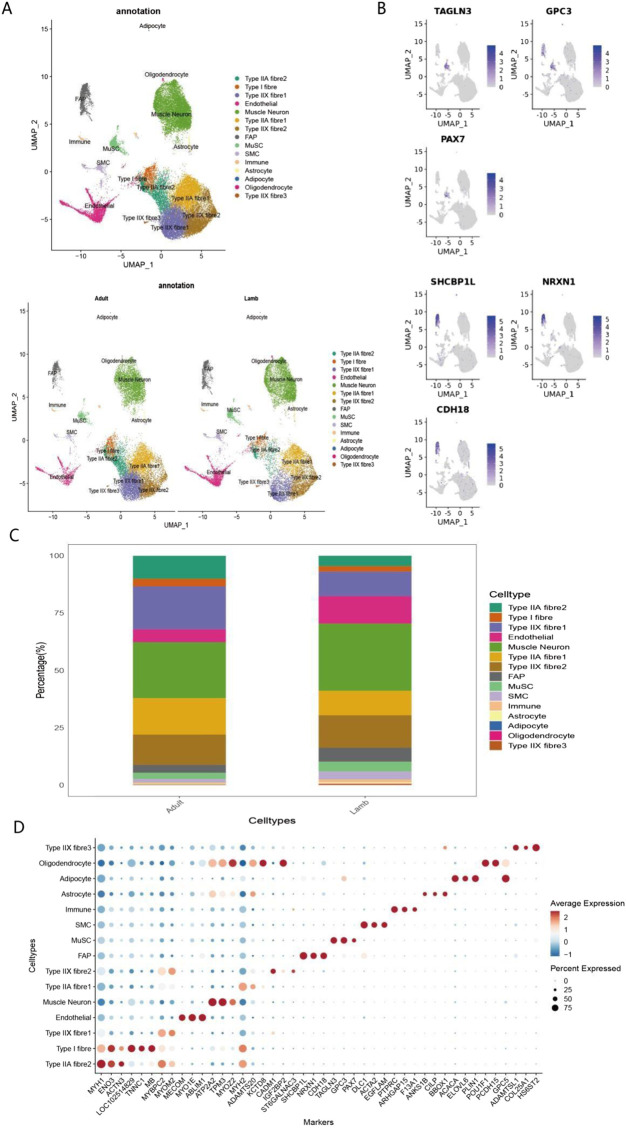
Single-cell transcriptomic analysis reveals cellular heterogeneity and compositional changes during skeletal muscle development in Bactrian camels **(A)** UMAP visualization showing the distribution characteristics of major cell types in Bactrian camel skeletal muscle. Different colors correspond to distinct cell clusters, including Type IIA fiber 2, Type I fiber, Type IIX fiber 1, endothelial cells, myoneurons, Type IIA fiber 1, Type IIX fiber 2, fibroblast activation protein-positive cells (FAPs), muscle satellite cells (MuSCs), smooth muscle cells (SMCs), immune cells, adipocytes, and oligodendrocytes. **(B)** UMAP visualization showing the expression patterns of selected marker genes (TAGLN3, GPC3, PAX7, SHCBP1L, NRXN1, and CDH10). Color intensity corresponds to gene expression levels (range: 0–4). **(C)** Stacked bar chart showing the relative proportions of cell types in skeletal muscle from adult and juvenile Bactrian camels. Different colors represent distinct cell types. **(D)** Dot plot showing the expression of cell type markers in the snRNA-seq dataset of Bactrian camel skeletal muscle. Dot size represents the proportion of cells expressing the marker; color intensity corresponds to the average expression level. High-expression patterns include: MYH7 in Type I muscle fibers, MYH2 in Type IIA muscle fibers, MYH1 in Type IIX muscle fibers, PECAM1/CDH5/CLDN5 in endothelial cells, PDGFRα/GSN in FAPs, ACTA2/MYH11 in SMCs, NRXN1/SNAP25 in neurons, GFAP/AQP4/ALDH1L1 in astrocytes, PLP1/MBP/MAG in oligodendrocytes, CD68/C1QA in immune cells, and PAX7/MYF5 in MuSCs.

Although numerous cell marker genes have been reported, their expression levels may vary due to factors such as species, age, and health status. To address this, the present study integrated recently published datasets of cell marker genes across multiple species, including humans, and specifically analyzed their expression characteristics in different cell populations of juvenile and adult Bactrian camels. Through single-cell sequencing and integrated clustering of longissimus dorsi muscle tissues from four Bactrian camels, a total of 14 cell subpopulations (clusters) were identified, primarily including: Muscle Neurons, Type IIx fibre cluster 1, Type IIx fibre cluster 2, Type IIx fibre cluster 3, Type IIa fibre cluster 1, Type IIa fibre cluster 2, Endothelial cells, fibro-adipogenic progenitors (FAPs), muscle satellite cells (MuSCs), smooth muscle cells (SMCs), Immune cells, Astrocytes, intramuscular Adipocytes, and Oligodendrocytes (Figures 1A,B). Separate analyses of longissimus dorsi muscle tissues from the two developmental stages revealed 14 cell clusters in each, but with significant differences in the proportions of each cell population. Compared to adult individuals, the muscle tissue of juvenile Bactrian camels exhibited distinct compositional characteristics: higher proportions of neurons, as well as relatively higher proportions of endothelial cells, fibro-adipogenic progenitors, muscle satellite cells, and smooth muscle cells. In contrast, the proportions of Type IIx fibre cluster 1, Type IIa fibre cluster 1, and Type IIa fibre cluster 2 in juvenile muscle tissue were all lower than those in adult individuals (Figure 1C). These results indicate that the proportions of different cell types undergo significant age-dependent changes. Transcriptomic analysis of Bactrian camel muscle development revealed significant shifts in cellular composition and function from the juvenile to the adult stage. Results showed that juvenile muscle tissue is enriched in supporting cells and stem cells, indicating robust growth and regenerative potential. Expression of the PAX7 and MYF5 genes plays key roles in maintaining the stemness of muscle satellite cells, laying the foundation for sustained growth. With advancing development, the proportions of mature Type IIx and IIa muscle fibres increase significantly. These fast-twitch fibres highly express genes such as MYH2, ACTA1, TNNT3, and TNNC2, which encode core contractile proteins crucial for muscle fibre function. Concurrently, the upregulation of PRKAR1A suggests that the cAMP-dependent protein kinase A signalling pathway plays an important role in regulating muscle metabolism and gene expression. This dynamic process reveals a developmental trajectory in which muscle tissue shifts from a growth-oriented to a functionally adaptive state. Complex gene expression networks orchestrate the balance between tissue growth, differentiation, and functional maturation, ultimately establishing muscle structures that meet the needs of adult animals.

### 3.2 Cell communication during the development of the longissimus muscle in bimodal hunchback

**FIGURE 2:**
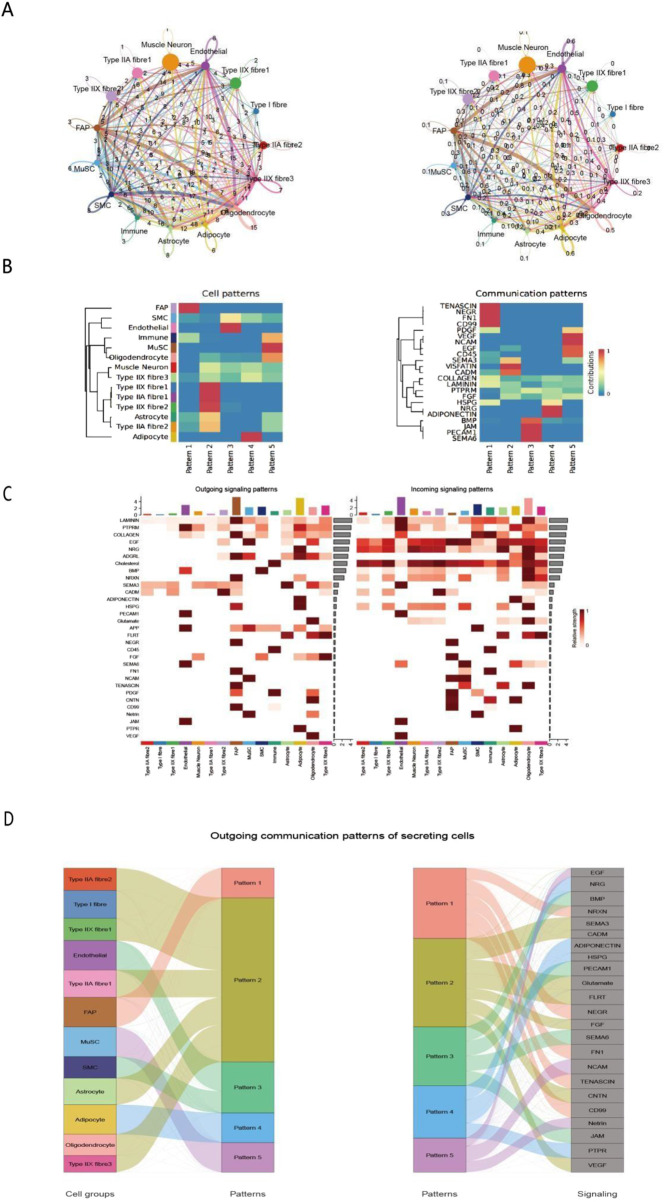
Analysis of intercellular communication networks and patterns in Bactrian camel skeletal muscle. **(A)** Circular network graph illustrating interactions between different cell types, including muscle neurons, endothelial cells, Type I/II/IIA/IIX muscle fibers, and supporting cells. Edge thickness represents the communication strength between nodes. **(B)**. Pattern analysis heatmap of cell types (left) and signaling pathways (right), showing the distribution characteristics of 5 patterns and the corresponding communication strength of signaling molecules (0–1, blue to red). **(C)**. Sankey diagram of secretory cell output communication patterns, illustrating the flow of signals from different cell types (left) through five communication patterns (middle) to different signaling molecules (right). Width of connecting bands indicates communication strength. **(D)** Sankey Diagram of Secreting Cells’ Outgoing Communication Patterns Left panel: associations between cell groups (e.g., Type IIA fibres, MuSCs) and communication patterns (1–5). Right panel: associations between these patterns and signaling molecules (e.g., EGF, VEGF). This reveals how secreting cells release specific signals via distinct patterns, aiding in dissecting their regulatory networks.

Based on our identification of distinct cell populations in Bactrian camels, we used the CellChat analytical approach (Liu et al., 2022) to construct an atlas of intercellular communication networks in the longissimus dorsi muscle. This network revealed complex signaling patterns among 12 cell populations (Figures 2A,B), including muscle neurons, three muscle fiber types (Type I, IIA, and IIX), and supporting cells. Analysis of ligand-receptor expression patterns showed that the strongest cellular interactions—with a signal strength > 8—occurred primarily among three key pairs: muscle neurons and Type IIA muscle fibers, endothelial cells and Type IIX muscle fibers, and fibroadipogenic progenitors (FAPs) and immune cells (Vitaliti et al., 2024). Our analysis of intercellular signaling networks revealed that a total of 27 key ligands/signaling molecules mediate communication among 13 cell subpopulations. Of particular note (Figure 2C), fibroblast activation protein-expressing (FAP) cells exhibited unique bidirectional regulatory properties. These cells transmit signals through two signaling pathways (Patterns 1 and 2), primarily secreting signaling molecules such as EGF (epidermal growth factor), HRG (heregulin), and ADGRL (adhesion G protein-coupled receptor L), while also showing high sensitivity to the HRG and BMP (bone morphogenetic protein) signaling pathways (Zhang et al., 2020). This bidirectional regulatory capacity enables FAP cells to act as core regulators in the tissue microenvironment, playing a key role in maintaining tissue homeostasis (Wang et al., 2022). Integrated analysis of cell patterns and signaling communication heatmaps further validated the complex signaling network constructed by FAP cells. This network comprises 20 key signaling molecules, encompassing growth factors, adhesion molecules, and extracellular matrix proteins, among other components, which can interact with multiple cell types (Figure 2D). These findings provide an important theoretical basis for understanding FAP cells in Bactrian camels.

### 3.3 Pseudotemporal trajectory analysis reveals relationships among different myogenic cell populations

**FIGURE 3:**
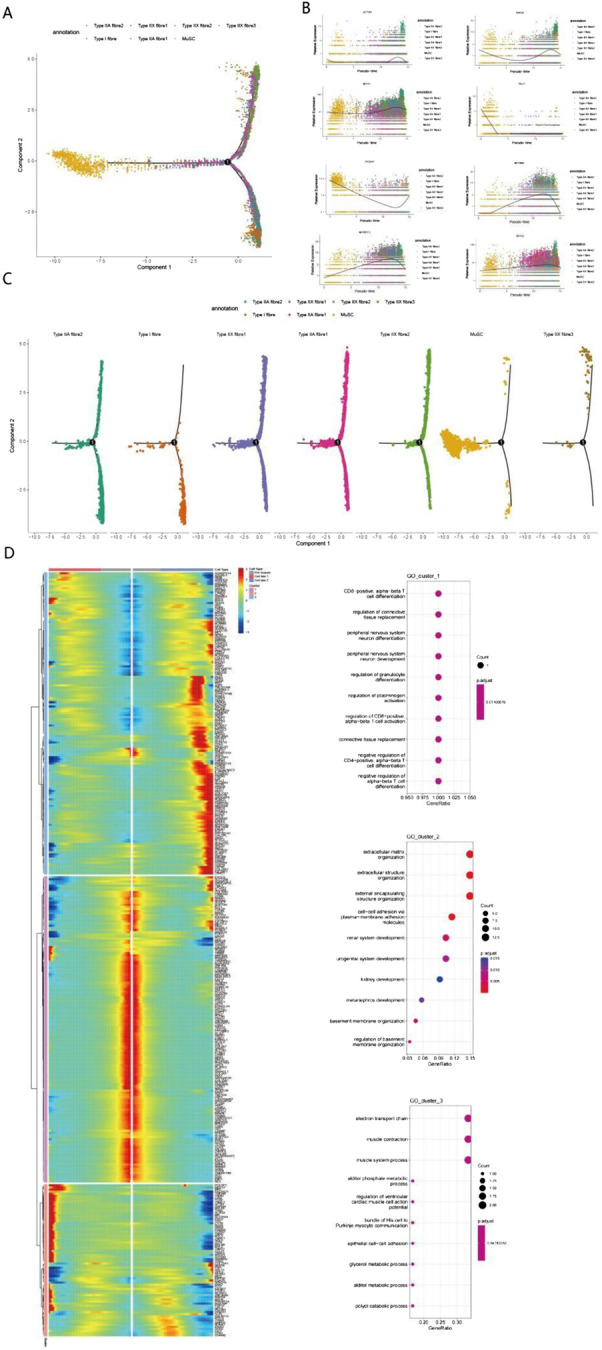
Single-cell transcriptomic analysis reveals multidimensional regulatory mechanisms of muscle fiber differentiation **(A)** Cell type distribution plot based on Principal Component Analysis (PCA) showing the spatial distribution characteristics of muscle fiber cells (including Type I, IIA, and IIX subtypes) and muscle stem cells. Cells exhibit a typical “Y”-shaped branching structure, extending leftward, upward, and downward from the origin, indicating multiple differentiation directions. **(B)** Pseudotime analysis reveals dynamic expression patterns of 8 key genes. The trend of gene expression levels over pseudotime illustrates the developmental progression of cells via color gradients. **(C)** Cell trajectory analysis clarifies the differentiation trajectories of different muscle fiber subtypes. **(D)** Gene expression clustering and functional enrichment analysis identify three major functional modules: T cell differentiation-related pathways, extracellular matrix and development, and muscle function and metabolism. Gene expression patterns are displayed via a heatmap; in the enrichment analysis, dot size and color represent the number of genes and statistical significance, respectively.

Pseudotime analysis results revealed the distribution characteristics of homotypic muscle fibers (Type I, IIA, and IIX) and muscle stem cells (MuSCs). At branch point 1, MuSCs underwent two distinct differentiation trajectories: one leading to fasttwitch muscle fibers (Type IIA, IIX), and the other differentiating into slow-twitch muscle fibers (Type I) as well as a MuSC subpopulation retaining stem cell properties (Figures 3A,C). During skeletal muscle development and fiber type differentiation, we observed sequential expression changes in several key genes. PAX7 (Figure 3B), the primary regulatory factor of satellite cells, showed high expression levels in early stages, consistent with its critical role in the fate determination of muscle stem cells (Sincennes et al., 2021). During differentiation, the expression patterns of MYH2 (Type IIA) and MYH1 (Type IIX) were closely associated with the formation of specific muscle fiber types; this expression regulatory network ultimately determines the contractile properties and metabolic characteristics of muscle fibers (Smith et al., 2023). With the progression of differentiation, genes related to muscle fiber type specificity showed differential expression patterns: ACTN3 and MYH1 were significantly upregulated in fast-twitch fibers, emerging as key factors determining the fast-twitch fiber phenotype (Oe et al., 2021). Increased expression of MYH2 and MYOM2 in late differentiation stages reflected the progressive maturation of muscle fiber structure, a phenomenon particularly pronounced in Type IIA fibers. The expression pattern of MYBPC2 indicated its important role in sarcomere assembly and the regulation of contractile function, with its expression levels closely associated with the metabolic characteristics of specific muscle fiber types (Lin et al., 2024).

**FIGURE 4:**
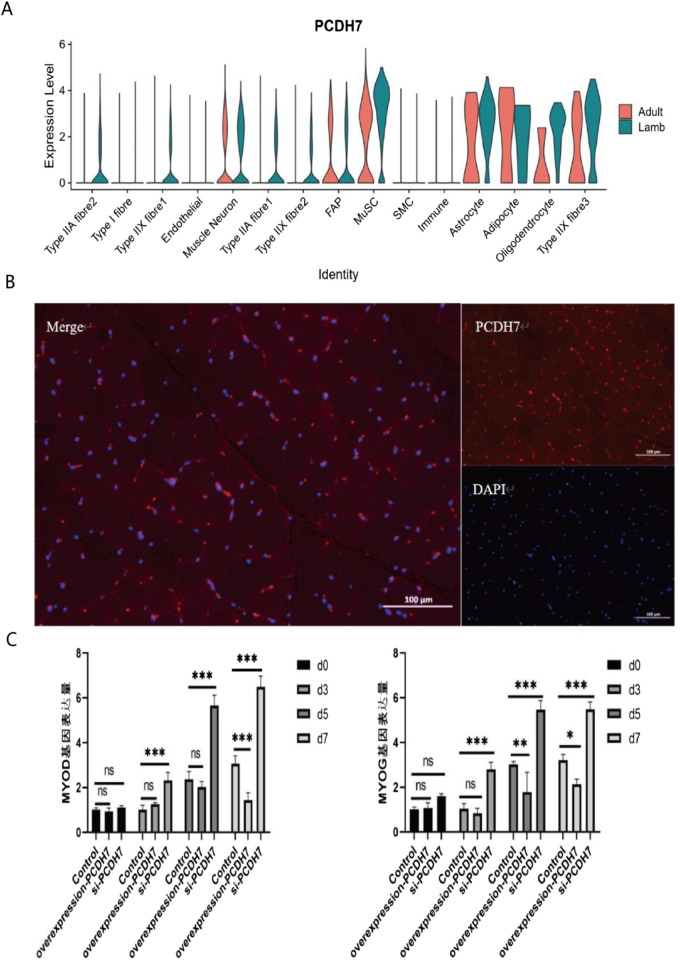
Expression characteristics and functional analysis of PCDH7 **(A)** Violin plot showing the expression distribution of PCDH7 across different cell types (including various fiber cells, neurons, immune cells, and glial cells) in adult and juvenile tissues. The y-axis represents expression levels, and the x-axis labels cell types. **(B)** Results of PCDH7 immunofluorescence localization. Left panel: Merged channel showing PCDH7 (red) and nuclear DAPI staining (blue); Top right: PCDH7 single-channel image; Bottom right: DAPI single-channel image. (Scale bar: 100 μm) **(C)** Temporal effects of PCDH7 expression on cell phenotype. Bar chart showing phenotypic changes in the control group and PCDH7 intervention group at different time points (Day 0, Day 3, Day 5, Day 7). Data are presented as mean ± standard deviation. ns indicates no significant difference; *P < 0.05; **P < 0.01; ***P < 0.001.

Notably, among the genes associated with longissimus dorsi muscle development identified via our pseudotime analysis, PCDH7—implicated in calcium ion binding and cell adhesion (Figures 4A,B; Wang et al., 2020)—has rarely been reported in studies on muscle tissue development in animal husbandry. To investigate the function of PCDH7, we performed overexpression and knockdown experiments in C2C12 cells. Results showed that PCDH7 significantly promotes myogenic differentiation: no significant differences were observed between groups during early differentiation stages (Days 0–3) (Figure 4C); as differentiation progressed, MYOG expression levels in the PCDH7 overexpression group increased significantly on Day 5 (P < 0.001) and remained high on Day 7, indicating that PCDH7 overexpression enhances myogenic differentiation. Conversely, in the PCDH7 knockdown group, MYOG expression levels decreased significantly from Day 3 (P < 0.01), and this inhibitory effect persisted until Days 5–7 (P < 0.05) (Figure 4C), suggesting that reduced PCDH7 expression impairs myogenic differentiation. Functional annotation of differentially expressed genes via GO enrichment analysis revealed three major functional clusters (Figure 3D). The first cluster primarily involves cell differentiation and tissue remodeling pathways, including regulation of CD8-positive T cell differentiation and mechanisms of connective tissue replacement. The second cluster shows significant enrichment in extracellular matrix organization and structural functions, particularly in the domains of extracellular matrix organization, extracellular structures, and cell adhesion molecules. The third cluster is mainly associated with muscle contraction and energy metabolism, encompassing key functions such as the electron transport chain, muscle contraction processes, and muscle system operation. Our findings indicate that a complex temporal regulatory network exists during Bactrian camel muscle development, spanning multiple levels including immune regulation, tissue remodeling, and muscle function development. Through systematic analysis, we revealed the pseudotemporal differentiation trajectory of myogenesis in Bactrian camels, confirming that this process exhibits unique spatiotemporal specificity and belongs to a heterogeneous cell differentiation mechanism.

### 3.4 MME^+^FAP cells are associated with adipogenesis

**FIGURE 5:**
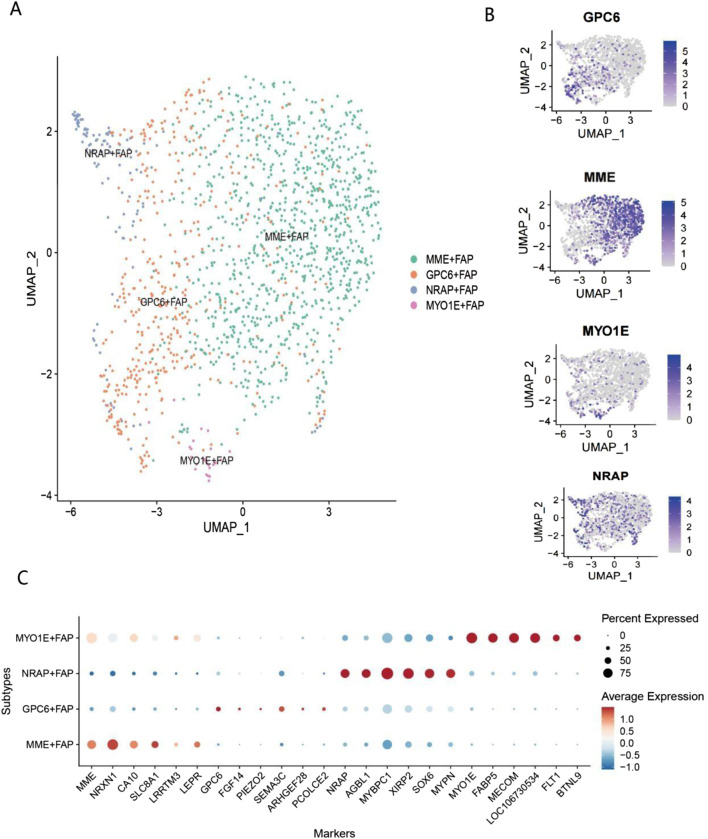
Single-cell transcriptomic analysis of FAP subpopulations **(A)** UMAP dimensionality reduction and clustering analysis of FAPs in Bactrian camel skeletal muscle. Different colors represent four distinct FAP subpopulations: MME-FAP (green), GPC6-FAP (orange), NRAP-FAP (blue), and MYO1E-FAP (purple). **(B)** Expression distribution of marker genes for each FAP subpopulation on UMAP plots. Expression patterns of GPC6, MME, MYO1E, and NRAP are shown from top to bottom. Intensity of purple indicates gene expression levels (0–5), with deep purple representing high expression and light gray representing low expression. **(C)** Dot plot of marker gene expression profiles in FAP subpopulations. The y-axis shows the four FAP subpopulations, and the x-axis displays 22 marker genes. The MME-FAP subpopulation highly expresses genes such as MME, NPXN, CAV1, S100A4, and LRRTM3; the GPC6-FAP subpopulation specifically expresses genes including GPC6, FBN2, and SFRP2; the NRAP-FAP subpopulation significantly expresses genes such as NRAP, MYH11, and ACTA2; and the MYO1E-FAP subpopulation specifically expresses genes including MYO1E, FABP4, and PECAM1.

In livestock production, intramuscular fat (IMF) content is one of the key indicators determining meat quality, with studies showing that it is significantly positively correlated with meat flavor, juiciness, and tenderness (Chen, 2024). Recent single-cell sequencing and lineage tracing studies have revealed that fibro-adipogenic progenitors (FAPs) are the primary source of intramuscular fat formation, and these cells participate in fat deposition through complex signaling network regulation, including the Wnt/β-catenin pathway (Liu, 2024; Reggio et al., 2020). Notably, FAPs exhibit significant multipotent characteristics, being capable of differentiating not only into adipocytes but also into myocytes and osteocytes; this multipotent differentiation potential is precisely regulated by epigenetic controls and microenvironmental factors (Zhang, 2024; Smith, 2024). Single-cell communication analysis results indicated that adipogenic progenitors (FAPs) exhibit significant signal interaction activity, making them a focus of subsequent research. Single-cell transcriptomic analysis revealed significant heterogeneity among adipogenic progenitors (FAPs) in Bactrian camel muscle tissue, with four major subpopulations identified: MME^+^FAP, GPC6^+^FAP, NRAP^+^FAP, and MYO1E^+^FAP (Figures 5A–C). Studies have shown that MME^+^FAPs, as a core adipogenic subpopulation, not only possess significant adipogenic differentiation potential but also play a key role in the process of muscle fat infiltration (Kang, 2023). The GPC6^+^FAP subpopulation enhances matrix production and tissue remodeling capabilities by secreting extracellular matrix proteins, thereby maintaining the structural homeostasis of muscle tissue (Theret, 2023). NRAP^+^FAPs are closely associated with muscle fiber regeneration, promoting the activation and differentiation of satellite cells by secreting various growth factors (Oprescu, 2023). The MYO1E^+^FAP subpopulation plays a key role in muscle damage repair, participating in inflammation regulation and tissue repair processes (Yin, 2024). It is particularly noteworthy that recent studies have suggested that FAP subpopulations can not only differentiate into white adipocytes but may also differentiate into brown or beige adipocytes under specific microenvironmental induction (Uezumi, 2023).

**FIGURE 6:**
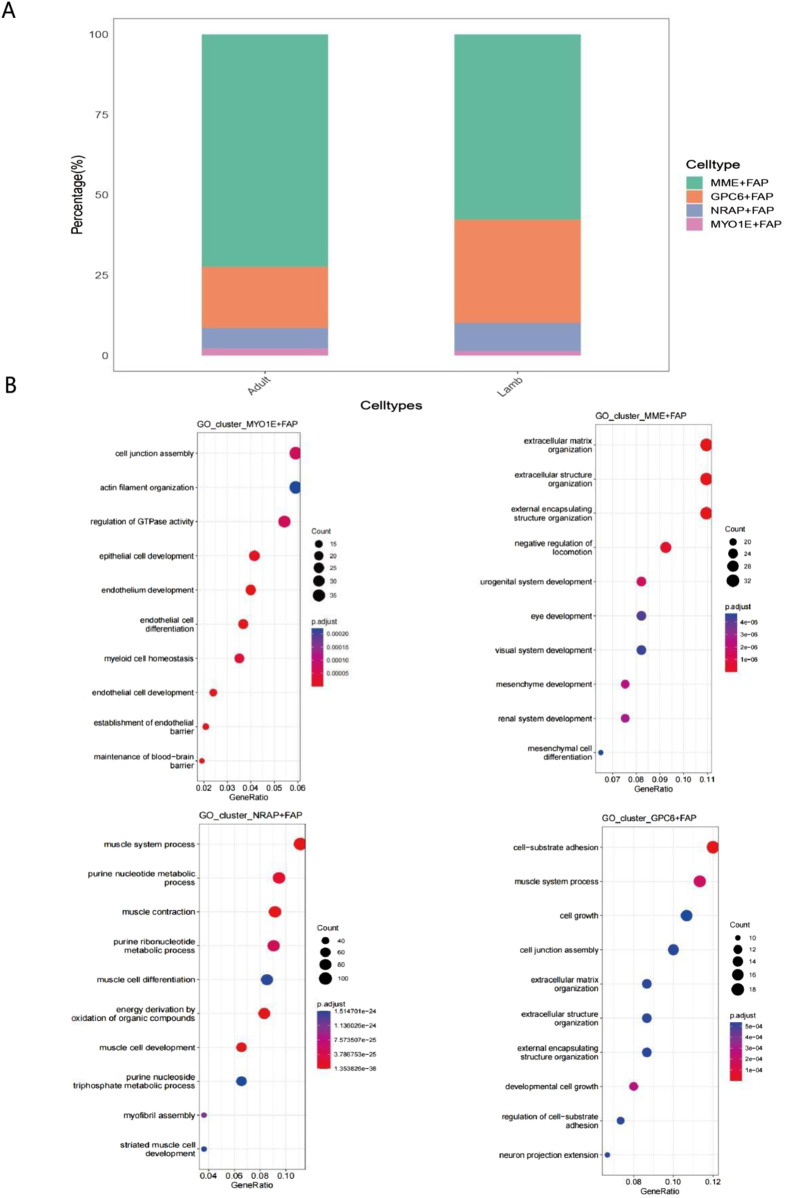
Analysis of the composition and functional characteristics of FAP subpopulations in adult and juvenile Bactrian camels **(A)** Stacked bar chart showing the relative proportions of different FAP subpopulations in adult and juvenile Bactrian camels. MME^+^ FAP represents the predominant population, while GPC6^+^ FAP significantly increases in juveniles. The y-axis indicates the percentage of each subpopulation, with different colors distinguishing the four FAP subtypes. **(B)** Bubble plot showing the results of GO functional enrichment analysis for the four FAP subpopulations. Bubble size represents the number of genes in each pathway; color intensity corresponds to the significance of p-values (darker colors indicate lower p-values). Each subpopulation has distinct functional characteristics: MYO1E^+^ FAP: enriched in cell junction-related pathways; MME^+^ FAP: enriched in extracellular matrix organization; GPC6^+^ FAP: enriched in cell-matrix adhesion; NRAP^+^ FAP: enriched in muscle system processes and metabolic processes.

In contrast, the proportion of the GPC6^+^FAP subpopulation was significantly higher in juveniles than in adults. The significant increase in the proportion of the MME^+^FAP subpopulation from the juvenile to the adult stage strongly supports its critical regulatory role in skeletal muscle maturation (Molina et al., 2021). Gene Ontology (GO) enrichment analysis revealed unique functional characteristics among these subpopulations. Four major FAP subpopulations were identified in both adult and juvenile Bactrian camels, among which the MME^+^FAP subpopulation was predominant in both age groups, with a significantly higher proportion in adults than in juveniles (Figure 6A). The MME^+^FAP subpopulation was primarily enriched in extracellular matrix (ECM)-related pathways (Figure 6B), including extracellular structure organization and external encapsulating structure organization. This subpopulation also showed significant enrichment in mesenchymal development and mesenchymal cell differentiation pathways, indicating its multifunctional roles in tissue remodeling and organ development (Fernández-Simón et al., 2024).

The GPC6^+^FAP subpopulation was significantly enriched in cell-matrix adhesion and muscle system processes, while also participating in cell junction assembly and developmental cell growth, collectively suggesting its key role in muscle fiber development and maintenance of tissue homeostasis. Notably, the NRAP^+^FAP subpopulation was highly enriched in muscle system processes and purine nucleoside metabolism. Its involvement in energy metabolism and oxidation of organic compounds indicates that this subpopulation plays an important role in muscle development through complex metabolic networks (Wang et al., 2024a). This subpopulation was also significantly enriched in functions related to myocyte differentiation and myofibril assembly. The MYO1E^+^FAP subpopulation was primarily involved in cell junction assembly and actin filament organization, while also enriched in pathways related to endothelial cell differentiation and blood-brain barrier establishment. This not only reflects its specific functions in muscle fiber development but also reveals its potential role in vascular development. Additionally, this subpopulation exhibited significant functions in epithelial cell development and regulation of GTPase activity, further supporting its multifunctional properties. This subpopulation-specific functional differentiation pattern provides new insights into the precise regulatory mechanisms of FAPs in muscle development and adipose deposition.

## 4 Discussion

For the first time, this study constructed a high-resolution cellular atlas of the longissimus dorsi muscle from juvenile (4 days old) and adult (5 years old) Bactrian camels using single-cell RNA sequencing (scRNA-seq), filling a gap in research on the molecular mechanisms of skeletal muscle development in Camelidae. A total of 14 cell clusters with distinct functional characteristics were identified, including muscle fibers, neurons, immune cells, and adipocytes, revealing age-dependent dynamic changes in cellular composition: Juvenile camel muscle tissue is dominated by muscle progenitor cells (e.g., muscle satellite cells, MuSCs) and stromal cells (e.g., endothelial cells), exhibiting growth-oriented features; in contrast, adult camel muscle is centered on mature muscle fibers (e.g., fast-twitch type IIX fibers) and neuromuscular junction cells, displaying functionally adaptive properties. This transition aligns closely with the desert survival strategy of Bactrian camels—Type IIX fibres in adult tissues, which rely on anaerobic metabolism and contract rapidly, facilitates short-term high-intensity activities in resource-scarce environments (Abdelhadi et al., 2012; Lyu et al., 2024; Kadim and Mahgoub, 2013), while the enrichment of progenitor cells in juveniles provides a developmental foundation for rapid muscle growth and adaptation to extreme environments (Son et al., 2021). This link between developmental trajectory and physiological function offers a unique desert species paradigm for understanding the adaptive evolution of mammalian skeletal muscle. Numerous studies have successfully identified cell-type-specific markers; however, it is critical to emphasize that the expression levels of these markers vary significantly across physiological stages. Our results revealed that type I muscle fibers are characterized by high expression of MYL3 and TNNC1, genes that play key roles in muscle fiber type determination and metabolic regulation (Wang et al., 2024b). Singlefiber proteomic analyses have shown that while type IIA and IIX fibers contain only trace amounts of MYL2, MYL3 is predominantly expressed in slow-twitch fibers (Murgia et al., 2021). This fiber-type-specific expression pattern, in coordination with other slow-twitch fiber markers (including TPM3 and TNNT1), forms a complex regulatory network governing muscle fiber type specialization and development (Deshmukh et al., 2019). Furthermore, recent transcriptomic analyses indicate that the expression levels of these genes—particularly TNNC1, TNNT2, and MYL3—are dynamically regulated during muscle development and show significant correlations with muscle fiber diameter and crosssectional area (Li et al., 2023). These findings not only validate the reliability of MYL3 and TNNC1 as specific markers for type I muscle fibers but also provide new insights into the molecular mechanisms underlying skeletal muscle fiber type determination and metabolic adaptation. Additionally, pseudotemporal trajectory analysis revealed the bidirectional differentiation fate of MuSCs: at developmental branch points, MuSCs can differentiate into slow-twitch type I fibers or fast-twitch type IIA/IIX fibers, and the cell adhesion molecule PCDH7 was identified as a novel regulator of myogenic differentiation. Studies have shown that PCDH7 plays a critical role in muscle development and atrophy, particularly in the progression of sarcopenia (Liu et al., 2023). The expression level of this gene also correlates significantly with carcass quality and meat traits in pigs (Belous et al., 2023). Our results demonstrate that PCDH7 promotes the expression of muscle development-related genes (MYOG, MYOD).

The findings of this study both align with the general rules of mammalian longissimus dorsi development and exhibit unique differences attributed to the ecological adaptability of Bactrian camels. In terms of commonalities, we confirmed that the multipotent differentiation potential of fibro-adipogenic progenitors (FAPs) is consistent with previous studies in mice and cattle (Li et al., 2020; Dohmen et al., 2022). FAPs regulate muscle repair and fat deposition through paracrine signaling pathways, and cell communication analysis further revealed their biological function as a signaling hub: FAPs interact with immune cells via the epidermal growth factor/heregulin (EGF/HRG) pathway, and mediate signal transmission with muscle fibers through the bone morphogenetic protein (BMP) pathway. This finding is consistent with conclusions from mouse models that FAPs maintain muscle homeostasis through paracrine mechanisms (Yin et al., 2024; Giuliani et al., 2022). However, species-specific differences are particularly notable. It is worth noting that in mouse models, the CD10^+^ (MME^+^) FAP subpopulation is primarily closely associated with muscle fibrosis progression (Schutz et al., 2024; Fitzgerald et al., 2023). As a type of mesenchymal progenitor cell with multipotent differentiation potential (Wang et al., 2024c), fibro-adipogenic progenitors (FAPs) can differentiate not only into fibroblasts and adipocytes (Uezumi et al., 2024) but also into osteoblasts and chondrocytes (Huang et al., 2024). Based on single-cell clustering analysis, this study further subdivided FAPs into four subpopulations: MME^+^, GPC6^+^, NRAP^+^, and MYO1E^+^. Among these, the proportion of the MME^+^FAP subpopulation increased significantly in the longissimus dorsi muscle of adult Bactrian camels. GO enrichment analysis showed that this subpopulation was significantly enriched in pathways related to extracellular matrix organization and mesenchymal cell differentiation. Existing studies have demonstrated that MME^+^ (CD10^+^) FAPs not only participate in adipogenesis by regulating adipogenic differentiation under physiological conditions (Schutz et al., 2024) but also regulate muscle regeneration and fibrosis through paracrine pathways (Molina et al., 2021). This subpopulation-specific functional differentiation pattern provides a new perspective for the regulatory mechanisms of longissimus dorsi homeostasis. Given that Bactrian camels are an important food source in arid regions, their intramuscular fat (IMF) content directly determines meat tenderness and flavor; thus, the identification of MME^+^FAPs provides a potential molecular target for the precision regulation of meat quality traits. In longissimus dorsi tissue, complex intercellular communication networks exhibit elaborate regulatory mechanisms. Muscle satellite cells (MuSCs) play a central role in muscle regeneration through mechanical memory and specific activation of signaling pathways (Madl et al., 2024). This study found extensive signal crosstalk between the three major muscle fiber types (I, IIA, and IIX) and niche cells, and these interactions are crucial for maintaining muscle tissue homeostasis (Wang et al., 2024d). Notably, FAPs and muscle cells form a bidirectional communication mechanism through specific signaling pathways, directly regulating the formation of intramuscular adipose tissue (IMAT) (Schmidt et al., 2023). In summary, this study constructed, for the first time, a single-cell transcriptomic atlas and intercellular communication network of the longissimus dorsi muscle in juvenile and adult Bactrian camels, revealing characteristics of cellular heterogeneity during muscle development. Notably, the analysis results indicated that MME^+^ (CD10^+^) adiposeassociated fibroblasts (FAPs) are significantly associated with adipogenesis. This study has certain limitations: On one hand, the sample size of Bactrian camels is limited (a total of 4 individuals were included, with 2 juveniles and 2 adults), which may impose certain restrictions on the generalizability of the study results; On the other hand, although the function of PCDH7 was verified using mouse C2C12 myotube cells, interspecies differences still warrant consideration, and the molecular pathway underlying fat production mediated by MME^+^(CD10^+^) fibroblasts (FAPs) in the longissimus dorsi muscle of camels also requires further investigation. Overall, this study provides important reference resources and a theoretical basis for the developmental mechanisms of Bactrian camel longissimus dorsi and the regulatory rules of meat quality traits.

## Data Availability

The data supporting the findings of this study related to the longissimus dorsi muscle of camels are openly available in the Genome Sequence Archive (GSA) hosted by the National Genomics Data Center (NGDC) at https://ngdc.cncb.ac.cn/gsub/submit/gsa/list accession number: CRA031969.

## References

[B1] Abdelhadi O. M. A. Babiker S. A. Picard B. Jurie C. Jailler R. Hocquette J. F. (2012). Effect of season on contractile and metabolic properties of desert camel muscle (*Camelus dromedarius*). Meat Sci. 90 (1), 139–144. 10.1016/j.meatsci.2011.06.012 21737209

[B2] Belous A. A. Sermyagin A. A. Zinovieva N. A. (2023). Genetic assessment of projected residual feed consumption and expression of significant candidate genes in duroc pigs and secondgeneration commercial blends. Russ. J. Genet. 59 (11), 1158–1172. 10.1134/s1022795423110029

[B3] Butler A. Hoffman P. Smibert P. Papalexi E. Satija R. (2018). Integrating single-cell transcriptomic data across different conditions, technologies, and species. Nat. Biotechnol. 36 (5), 411–420. 10.1038/nbt.4096 29608179 PMC6700744

[B4] Chen X. (2024). Advances in understanding intramuscular fat development and meat quality regulation. Nat. Food 5, 156–170.

[B5] Cheng W. Cheng J. H. Sun D. W. Pu H. (2015). Marbling analysis for evaluating meat quality: methods and techniques. Compr. Rev. Food Sci. Food Saf. 14 (5), 523–535. 10.1111/1541-4337.12149

[B6] Deshmukh A. S. Murgia M. Nagaraj N. Treebak J. T. Cox J. Mann M. (2019). Deep proteomics of mouse skeletal muscle enables quantitation of protein isoforms, metabolic pathways, and transcription factors. Mol. and Cell. Proteomics 18 (5), 841–853. 10.1074/mcp.M114.044222 25616865 PMC4390264

[B7] Dohmen R. G. J. Hubalek S. Melke J. Messmer T. Cantoni F. Mei A. (2022). Muscle-derived fibro-adipogenic progenitor cells for production of cultured bovine adipose tissue. NPJ Sci. Food 6 (1), 6. 10.1038/s41538-021-00122-2 35075125 PMC8786866

[B8] Fernández-Simón E. Piñol-Jurado P. Gokul-Nath R. Unsworth A. Alonso-Pérez J. Schiava M. (2024). Single cell RNA sequencing of human FAPs reveals different functional stages in Duchenne muscular dystrophy. Front. Cell Dev. Biol. 12, 1399319. 10.3389/fcell.2024.1399319 39045456 PMC11264872

[B9] Fitzgerald G. Turiel G. Gorski T. (2023). MME+ fibro-adipogenic progenitors are the dominant adipogenic population during fatty infiltration in human skeletal muscle. Commun. Biol. 6 (1), 111. 36707617 10.1038/s42003-023-04504-y PMC9883500

[B10] Giuliani G. Rosina M. Reggio A. (2022). Signaling pathways regulating the fate of fibro/adipogenic progenitors (FAPs) in skeletal muscle regeneration and disease. FEBS J. 289 (21), 6484–6517. 10.1111/febs.16080 34143565

[B11] Hafemeister C. Satija R. (2019). Normalization and variance stabilization of single-cell RNAseq data using regularized negative binomial regression. Genome Biol. 20 (1), 296. 10.1186/s13059-019-1874-1 31870423 PMC6927181

[B12] Hao Y. Hao S. Andersen-Nissen E. (2024). Integrated analysis of multimodal single-cell data. Cell 187 (1), 177–194.e17. 10.1016/j.cell.2021.04.048 PMC8238499 34062119

[B13] Huang Y. Wang X. Lin H. (2024). Cellular and molecular mechanisms of heterotopic ossification in skeletal muscle. Int. J. Mol. Sci. 25(7), 3641. 38612455

[B14] Jin L. Tang Q. Hu S. Chen Z. Zhou X. Zeng B. (2021a). A pig BodyMap transcriptome reveals diverse tissue physiologies and evolutionary dynamics of transcription. Nat. Commun. 12 (1), 3715. 10.1038/s41467-021-23560-8 34140474 PMC8211698

[B15] Jin S. Guerrero-Juarez C. F. Zhang L. Chang I. Ramos R. Kuan C. H. (2021b). Inference and analysis of cell-cell communication using CellChat. Nat. Commun. 12 (1), 1088. 10.1038/s41467-021-21246-9 33597522 PMC7889871

[B16] Jones R. C. Karkanias J. Krasnow M. A. Pisco A. O. Quake S. R. Salzman J. (2022). The tabula sapiens: a multiple- organ, single-cell transcriptomic atlas of humans. Science 376 (6594), eabl4896. 10.1126/science.abl4896 35549404 PMC9812260

[B17] Jovic D. Liang X. Zeng H. Lin L. Xu F. Luo Y. (2022). Single-cell RNA sequencing technologies and applications: a brief overview. Clin. Transl. Med. 12 (4), e694. 10.1002/ctm2.694 35352511 PMC8964935

[B18] Kadim I. T. Mahgoub O. (2013). Structure and quality of camel meat[M]//Camel meat and meat products. Wallingford UK: CABI, 124–152.

[B19] Kadim I. T. Al-Amri I. S. Al Kindi A. Y. (2018). Camel meat production and quality: a review. J. Camel Pract. Res. 25 (1), 1–11.

[B20] Kang X. (2023). MME+ fibro-adipogenic progenitors are the dominant adipogenic population in human skeletal muscle. Commun. Biol. 6, 115. 36707617 10.1038/s42003-023-04504-y PMC9883500

[B21] Kang W. Xie Q. Ma L. (2023). Integration and batch effect correction enable robust cell type identification in high-dimensional single-cell datasets. Nat. Commun. 14 (1), 2999. 37225702

[B22] Li X. Fu X. Yang G. Du M. (2020). Review: enhancing intramuscular fat development *via* targeting fibro-adipogenic progenitor cells in meat animals. Animal 14 (2), 312–321. 10.1017/S175173111900209X 31581971

[B23] Li H. Zhao X. Ding X. (2023). Transcriptome analysis reveals the molecular mechanisms underlying muscle fiber type determination and growth in chickens. Poult. Sci. 102 (12), 103088. 37741119

[B24] Lin Y. Sun L. Lv Y. Liao R. Zhang K. Zhou J. (2024). Transcriptomic and metabolomic dissection of skeletal muscle of crossbred chongming white goats with different meat production performance. BMC Genomics 25 (1), 443–15. 10.1186/s12864-024-10304-3 38704563 PMC11069289

[B25] Liu J. (2024). Single-cell analysis reveals developmental trajectories of fibro-adipogenic progenitors in muscle. Cell Stem Cell 29, 234–248.

[B26] Liu Z. Sun D. Wang C. (2022). Evaluation of cell-cell interaction methods by integrating singlecell RNA sequencing data with spatial information. Genome Biol. 23 (1), 218. 10.1186/s13059-022-02783-y 36253792 PMC9575221

[B27] Liu M. Wang Y. Shi W. Yang C. Wang Q. Chen J. (2023). PCDH7 as the key gene related to the co-occurrence of sarcopenia and osteoporosis. Front. Genet. 14, 1163162. 10.3389/fgene.2023.1163162 37476411 PMC10354703

[B28] Luecken M. D. Theis F. J. (2019). Current best practices in single‐cell RNA‐seq analysis: a tutorial. Mol. Syst. Biol. 15 (6), e8746. 10.15252/msb.20188746 31217225 PMC6582955

[B29] Lyu H. Na Q. Wang L. Li Y. Zheng Z. Wu Y. (2024). Effects of muscle type and aging on glycolysis and physicochemical quality properties of bactrian camel (*Camelus bactrianus*) meat. Animals 14 (4), 611. 10.3390/ani14040611 38396579 PMC10886407

[B30] Madl C. M. Wang Y. X. Holbrook C. A. Su S. Shi X. Byfield F. J. (2024). Hydrogel biomaterials that stiffen and soften on demand reveal that skeletal muscle stem cells harbor a mechanical memory. Proc. Natl. Acad. Sci. 121 (35), e2406787121. 10.1073/pnas.2406787121 39163337 PMC11363279

[B31] Molina T. Fabre P. Dumont N. A. (2021). Fibro-adipogenic progenitors in skeletal muscle homeostasis, regeneration and diseases. Open Biol. 11 (12), 210110. 10.1098/rsob.210110 34875199 PMC8651418

[B32] Mu Q. Chen Y. Wang J. (2019). Deciphering brain complexity using single-cell sequencing. Genomics Proteomics Bioinforma. 17 (4), 344–366. 10.1016/j.gpb.2018.07.007 31586689 PMC6943771

[B33] Murgia M. Toniolo L. Nagaraj N. (2021). Single muscle fiber proteomics reveals fiber-type-specific features of human muscle aging. Cell Rep. 32 (1), 107893. 10.1016/j.celrep.2017.05.054 28614723

[B34] Oe M. Ojima K. Muroya S. (2021). Difference in potential DNA methylation impact on gene expression between fast-and slow-type myofibers. Physiol. Genomics 53 (2), 69–83. 10.1152/physiolgenomics.00099.2020 33459151

[B35] Oprescu S. N. (2023). Thrown for a loop: fibro-adipogenic progenitors in skeletal muscle regeneration and disease. Am. J. Physiology-Cell Physiology 325, C1C16. 10.1152/ajpcell.00245.2023 PMC11932532 37602412

[B36] Potter S. S. (2018). Single-cell RNA sequencing for the study of development, physiology and disease. Nat. Rev. Nephrol. 14 (8), 479–492. 10.1038/s41581-018-0021-7 29789704 PMC6070143

[B37] Qiu X. Hill A. Packer J. Lin D. Ma Y. A. Trapnell C. (2017a). Single-cell mRNA quantification and differential analysis with census. Nat. Methods 14 (3), 309–315. 10.1038/nmeth.4150 28114287 PMC5330805

[B38] Qiu X. Mao Q. Tang W. Wang L. Chawla R. Pliner H. A. (2017b). Reversed graph embedding resolves complex single-cell trajectories. Nat. Methods 14 (10), 979–982. 10.1038/nmeth.4402 28825705 PMC5764547

[B39] Reggio A. Rosina M. Palma A. Cerquone Perpetuini A. Petrilli L. L. Gargioli C. (2020). Adipogenesis of skeletal muscle fibro/adipogenic progenitors is affected by the WNT5a/GSK3/β-catenin axis. Cell Death and Differ. 27 (10), 2921–2941. 10.1038/s41418-020-0551-y 32382110 PMC7492278

[B40] Schmidt M. Schuler S. C. Hüttner S. S. (2023). Adult muscle stem cells: insights on molecular mechanisms influencing their fate decisions. Nat. Commun. 14 (1), 1–19. 36596776

[B41] Schutz P. W. Cheung S. Yi L. Rossi F. M. V. (2024). Cellular activation patterns of CD10+ fibroadipogenic progenitors across acquired disease states in human skeletal muscle biopsies. Free Neuropathol. 5, 3. 10.17879/freeneuropathology-2024-5162 38357523 PMC10865694

[B42] Sincennes M. C. Brun C. E. Lin A. Y. T. Rosembert T. Datzkiw D. Saber J. (2021). Acetylation of PAX7 controls muscle stem cell self-renewal and differentiation potential in mice. Nat. Commun. 12 (1), 3253. 10.1038/s41467-021-23577-z 34059674 PMC8167170

[B43] Smith K. R. (2024). Molecular mechanisms controlling FAP fate decisions in livestock animals. Cell and Biosci. 14, 45.

[B44] Smith J. A. B. Murach K. A. Dyar K. A. Zierath J. R. (2023). Exercise metabolism and adaptation in skeletal muscle. Nat. Rev. Mol. Cell Biol. 24 (9), 607–632. 10.1038/s41580-023-00606-x 37225892 PMC10527431

[B45] Son Y. B. Jeong Y. I. Jeong Y. W. Hossein M. S. Olsson P. O. Tinson A. (2021). Cell source-dependent *in vitro* chondrogenic differentiation potential of mesenchymal stem cell established from bone marrow and synovial fluid of *Camelus Dromedarius* . Animals 11 (7), 1918. 10.3390/ani11071918 34203207 PMC8300404

[B46] Stuart T. Butler A. Hoffman P. Hafemeister C. Papalexi E. Mauck W. M. (2019). Comprehensive integration of single-cell data. Cell 177 (7), 1888–1902. 10.1016/j.cell.2019.05.031 31178118 PMC6687398

[B47] TABULA MURIS CONSORTIUM (2020). A single-cell transcriptomic atlas characterizes ageing tissues in the mouse. Nature 583 (7817), 590–595. 10.1038/s41586-020-2496-1 32669714 PMC8240505

[B48] The Tabula Muris Consortium, Overall coordination, Logistical coordination, Organ collection and processing, Library preparation and sequencing, Computational data analysisOverall coordinationLogistical coordinationOrgan collection and processingLibrary preparation and sequencingComputational data analysis (2018). Single-cell transcriptomics of 20 mouse organs creates a tabula muris. Nature 562, 367–372. 10.1038/s41586-018-0590-4 30283141 PMC6642641

[B49] Theret M. (2023). Identification and isolation of a unique skeletal muscle resident mesenchymal stem cell population with distinct differentiation and immunomodulatory capacities. Nat. Cell Biol. 25, 1321–1337.

[B50] Uezumi A. (2023). Mesenchymal progenitors distinct from satellite cells contribute to ectopic fat cell formation in skeletal muscle. Nat. Cell Biol. 25, 1338–1352. 10.1038/ncb2014 20081842

[B51] Uezumi A. Ikemoto-Uezumi M. Zhou H. (2024). Involvement of lysophosphatidic acid-LPA1-YAP signaling in healthy and pathological skeletal muscle. Matrix Biol. 129, 101481. 10.1016/j.matbio.2024.08.005 39153517

[B52] Van Ba H. Oliveros C. M. Park K. M. Dashdorj D. Hwang I. (2016). Effect of marbling and chilled ageing on meatquality traits, volatile compounds and sensory characteristics of beef longissimus dorsi muscle. Animal Prod. Sci. 57 (5), 981–992. 10.1071/an15676

[B53] Vitaliti A. Reggio A. Colletti M. Galardi A. Palma A. (2024). Integration of single-cell datasets depicts profiles of macrophages and fibro/adipogenic progenitors in dystrophic muscle. Exp. Cell Res. 442 (1), 114197. 10.1016/j.yexcr.2024.114197 39111382

[B54] Wang C. Chen A. Ruan B. Niu Z. Su Y. Qin H. (2020). PCDH7 inhibits the formation of homotypic cell-in-cell structure. Front. Cell Dev. Biol. 8, 329. 10.3389/fcell.2020.00329 32457908 PMC7225324

[B55] Wang Y. Zhang L. Wu G. (2022). Single-cell transcriptomics reveals regulators of muscle stem cell function. Cell Stem Cell 29 (8), 1206–1222.

[B56] Wang L. Zhou Y. Wang Y. Shan T. (2024a). Integrative cross-species analysis reveals conserved and unique signatures in fatty skeletal muscles. Sci. Data 11 (1), 290. 10.1038/s41597-024-03114-5 38472209 PMC10933306

[B57] Wang Y. Zhang X. Wang Z. (2024b). Differential expression of myosin light chain genes in different muscle fiber types of beef cattle. Animals 14 (15), 2225.39123750

[B58] Wang L. Zhang C. Zhang Y. (2024c). FAPs orchestrate homeostasis of muscle physiology and regeneration. FASEB J. 38 (4), e202400381. 10.1096/fj.202400381R PMC11647758 39676717

[B59] Wang Y. Zhang X. Huang H. Heeren A. A. White T. A. Li H. (2024d). Senescent skeletal muscle fibroadipogenic progenitors recruit and promote M2 polarization of macrophages. Aging Cell 23 (3), e14069. 10.1111/acel.14069 38115574 PMC10928562

[B60] Xu D. Wan B. Qiu K. Wang Y. Zhang X. Jiao N. (2023a). Single-cell RNA-sequencing provides insight into skeletal muscle evolution during the selection of muscle characteristics. Adv. Sci. 10, e2305080. 10.1002/advs.202305080 37870215 PMC10724408

[B61] Xu J. Liang Y. Chen Y. (2023b). Systematic comparison and evaluation of scRNA-seq batch effect removal tools in cancer studies. Mol. Cancer 22 (1), 41.36859185

[B62] Xu S. Hu E. Cai Y. Xie Z. Luo X. Zhan L. (2024). Using clusterProfiler to characterize multiomics data. Nat. Protoc. 19, 3292–3320. 10.1038/s41596-024-01020-z 39019974

[B63] Yang X. Zhou Y. Jin R. (2023). Single-cell RNA-Seq drives the construction of human cell landscape and disease mechanisms. Cell Death Discov. 9 (1), 61. 36781845

[B64] Yin H. (2024). MuSCs and IPCs: roles in skeletal muscle homeostasis, aging and diseases. Cell. Mol. Life Sci. 81, 31. 38289345 10.1007/s00018-023-05096-w PMC10828015

[B65] Yin K. Zhang C. Deng Z. Wei X. Xiang T. Yang C. (2024). FAPs orchestrate homeostasis of muscle physiology and pathophysiology. FASEB J. 38 (24), e70234. 10.1096/fj.202400381R 39676717 PMC11647758

[B66] Zhang Y. (2024). Epigenetic regulation of FAP multipotency in skeletal muscle development. Sci. Adv. 10, eadf8721.

[B67] Zhang L. Wang Y. Xiao F. (2020). Single-cell analysis of the metabolic landscape of skin fibroblasts reveals therapeutic vulnerabilities in systemic sclerosis. Nat. Metab. 2 (9), 898–911.

[B68] Zheng G. X. Terry J. M. Belgrader P. Ryvkin P. Bent Z. W. Wilson R. (2017). Massively parallel digital transcriptional profiling of single cells. Nat. Commun. 8, 14049. 10.1038/ncomms14049 28091601 PMC5241818

